# Mitochondrial GpC and CpG DNA Hypermethylation Cause Metabolic Stress-Induced Mitophagy and Cholestophagy

**DOI:** 10.3390/ijms242216412

**Published:** 2023-11-16

**Authors:** Claudia Theys, Joe Ibrahim, Ligia Mateiu, Archibold Mposhi, Laura García-Pupo, Tim De Pooter, Peter De Rijk, Mojca Strazisar, İkbal Agah İnce, Iuliana Vintea, Marianne G. Rots, Wim Vanden Berghe

**Affiliations:** 1 Lab Protein Chemistry, Proteomics & Epigenetic Signaling (PPES), Department Biomedical Sciences, University of Antwerp, Wilrijk, 2610 Antwerp, Belgium; claudia.theys@uantwerpen.be (C.T.);; 2 Center of Medical Genetics, University of Antwerp and Antwerp University Hospital, 2650 Edegem, Belgium; 3 Center for Oncological Research, University of Antwerp and Antwerp University Hospital, 2650 Edegem, Belgium; 4 Department of Pathology and Medical Biology, University of Groningen, University Medical Center Groningen, 9713 GZ Groningen, The Netherlands; 5 Neuromics Support Facility, VIB Center for Molecular Neurology, VIB, Wilrijk, 2610 Antwerp, Belgium; 6 Department of Biomedical Sciences, University of Antwerp, Wirlijk, 2610 Antwerp, Belgium; 7 Department of Medical Microbiology, School of Medicine, Acıbadem Mehmet, Ali Aydınlar University, 34752 Ataşehir, İstanbul, Türkiye; 8 Pathophysiology Lab, Infla-Med Centre of Excellence, Department of Biomedical Sciences, University of Antwerp, Wilrijk, 2610 Antwerp, Belgium

**Keywords:** mitochondrial epigenetics, MASLD, lipid metabolism, bile acid metabolism, cholestasis, autophagy

## Abstract

Metabolic dysfunction-associated steatotic liver disease (MASLD) is characterized by a constant accumulation of lipids in the liver. This hepatic lipotoxicity is associated with a dysregulation of the first step in lipid catabolism, known as beta oxidation, which occurs in the mitochondrial matrix. Eventually, this dysregulation will lead to mitochondrial dysfunction. To evaluate the possible involvement of mitochondrial DNA methylation in this lipid metabolic dysfunction, we investigated the functional metabolic effects of mitochondrial overexpression of CpG (MSssI) and GpC (MCviPI) DNA methyltransferases in relation to gene expression and (mito)epigenetic signatures. Overall, the results show that mitochondrial GpC and, to a lesser extent, CpG methylation increase bile acid metabolic gene expression, inducing the onset of cholestasis through mito-nuclear epigenetic reprogramming. Moreover, both increase the expression of metabolic nuclear receptors and thereby induce basal overactivation of mitochondrial respiration. The latter promotes mitochondrial swelling, favoring lipid accumulation and metabolic-stress-induced mitophagy and autophagy stress responses. In conclusion, both mitochondrial GpC and CpG methylation create a metabolically challenging environment that induces mitochondrial dysfunction, which may contribute to the progression of MASLD.

## 1. Introduction

Metabolic dysfunction-associated steatotic liver disease (MASLD) is a growing epidemic, which mirrors the increased trend of obesity in Western diet-consuming countries. It has an estimated prevalence of 20–30% in Europe and is the most common cause of chronic liver disease worldwide [1,2]. MASLD consists of a spectrum of liver disorders ranging from simple steatosis to metabolic dysfunction-associated steatohepatitis (MASH), which predisposes patients to further fibrosis, cirrhosis, and hepatocarcinoma, as well as extrahepatic diseases, especially cardiovascular diseases [3,4]. Despite the increasing prevalence, there is still no FDA-approved treatment for MASLD. A change in lifestyle, including a restricted diet and an increase in exercise, is currently the only approved therapy. However, this treatment is difficult to maintain, which causes relapse in a lot of patients [5,6]. Therefore, a lot of research has been focused on the identification of new therapeutic targets.

There are several risk factors for the development of MASLD, including the environment, genetics, and epigenetics. It is known that environmental factors, including diet and pollutants, are related to an increased risk of the development of MASLD [7]. In addition, different mutants have been associated with an increased risk for MASLD development, with mutations in the *PNPLA3* gene being one of the main genetic risk factors [7,8]. However, neither environmental factors nor genetic factors alone can give a satisfactory explanation for the high prevalence of MASLD. Therefore, researchers are now also searching for epigenetic factors contributing to the development and progression of MASLD. Interestingly, epigenetic modifications are both related to environmental exposures and genetic predispositions. These modifications include DNA methylation, histone modifications, and miRNAs [9], although most of the epigenetic studies in MASLD patients have mainly focused on DNA methylation, revealing MASLD stage-dependent signatures [10,11,12,13,14,15,16,17]. Besides nuclear DNA methylation patterns, MASLD-specific methylation changes have recently also been reported in mitochondrial DNA [18,19,20,21].

Mitochondria are critically involved in MASLD progression because fatty acid beta oxidation takes place in the mitochondrial matrix, which is part of lipid catabolism [22]. Hence, in the steatosis stage, hepatocytes try to overcome excess lipid accumulation by increasing beta oxidation in the mitochondria. This mitochondrial hyperactivation can result in oxidative mitochondrial damage and eventually a complete metabolic shutdown due to mitochondrial dysfunction. The latter is closely linked to MASH, showing less active mitochondria and more mitochondrial stress, which is a contributing factor to other complications including inflammation and fibrosis [23,24]. Whether epigenetic modifications are able to fine-tune mitochondrial metabolic respiratory functions following lipid-induced stress has not yet been functionally addressed. Interestingly, DNA methyltransferase 1 (DNMT1) can translocate into the mitochondria and thereby contribute to the hypermethylation of the mtDNA during MASLD progression because MASH patients show an increased expression of DNMT1 [25,26]. Indeed, Pirola et al. and Mposhi et al. showed that MASH patients have increased methylation of the *ND6* region of the mitochondrial DNA, which resulted in a downregulation of ND6 expression [21,26]. Mposhi et al. further functionally confirmed these findings by bisulfite pyrosequencing, qPCR, and nanopore-episequencing approaches in a mouse model and in transgenic steatosis HepG2 cell models with mitochondrial overexpression of CpG (MSssI) or GpC (MCviPI)-specific DNA methyltransferases, which similarly revealed evidence for MASH-specific lipid metabolic changes in gene expression [21]. Following up on these observations, we here applied a systems biology approach to further address the functional contribution of mitochondrial DNA methylation in metabolic dysfunctions during MASLD-associated lipid accumulation stress. More particularly, to resolve mito-nuclear epigenetic crosstalk associated with mitochondrial DNA methylation and functional mitochondrial changes in morphology, respiratory activity, and metabolic competence, we performed an in-depth integrative genome-wide transcriptome-epigenome analysis.

## 2. Results

### 2.1. Overexpression of Mitochondrial Targeted DNMTs Promotes GpC and CpG mtDNA Hypermethylation

**Figure 1:**
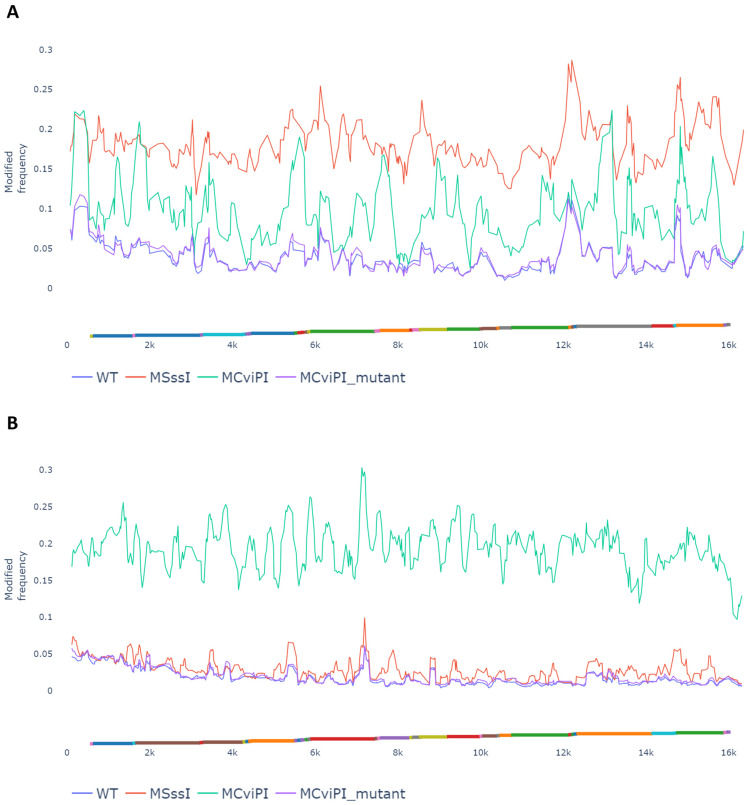
Mitochondrial MCviPi or MSssI overexpression increases mitochondrial GpC or CpG methylation, respectively. Modified frequency showing the per read per position frequency of CpG (**A**) or GpC (**B**) methylation in the mitochondrial genome of the different cell lines. WT and MCviPI mutants are both control cell lines with no expression (untransfected) or overexpression of an inactive DNMT (mock transfected), respectively. MCviPI overexpresses a GpC DNMT targeted to the mitochondrial genome; MSssI overexpresses a CpG DNMT targeted to the mitochondrial genome. Mitochondrial genes are represented as different colors on the *x*-axis.

Mitochondria play a crucial role in lipid catabolism because beta oxidation is regulated in the mitochondrial matrix [22]. Thus, it is not surprising that mitochondrial dysfunction is involved in lipid metabolic disorders such as MASLD. Mposhi et al. and Pirola et al. reported that the mitochondrial *ND6* region, an essential component of Complex I of the OXPHOS cycle, is hypermethylated and downregulated in cell lines overexpressing a mitochondrial targeted CpG-specific DNMT (MSssI) or a GpC-specific DNMT (MCviPI) and MASH patients compared to patients with steatosis [21,26]. To further dissect how mitochondrial DNA methylation promotes the onset and progression of MASLD in the latter in vitro steatosis model with mitochondrial CpG and GpC DNA methyltransferases, we combined genome-wide transcriptomic-epigenomic systems biology approaches with functional mitochondrial assays to compare morphology, respiratory activity, and metabolic competence in the different setups. First, changes in mitochondrial CpG and GpC DNA methylation levels were confirmed by Nanopore episequencing in the MCviPI and MSssI DNMT overexpressing HepG2 cell lines, showing specifically increased CpG and GpC mtDNA methylation versus low baseline mtDNA methylation levels in the mock transfected MCviPI DNMT mutant and un-transfected (WT) HepG2 cell lines, as observed earlier by Mposhi et al. [21] (Figure 1). Generally, there was a coverage of 60–80× of the mitochondrial DNA, which is sufficient for qualitative and quantitative mapping of mitochondrial methylation changes. The untransfected naive HepG2 cell line (WT), which lacks DNMT overexpression, and the HepG2 cell line, which overexpresses a MCviPI-deficient DNMT (MCviPI mutant), were both used as baseline reference mtDNA methylation control cell lines (mean methylation frequency ± SD: WT—CpG Me 0.040 ± 0.04 or 4.0 ± 0.4%, MCviPI mutant CpG Me 0.041 ± 0.04 or 4.1 ± 0.4%; WT GpC Me 0.019 ± 0.02 or 1.9 ± 0.2%, MCviPI mutant GpC 0.021 ± 0.02 or 2.1 ± 0.2%). This reveals that baseline CpG methylation is slightly more abundant than GpC mtDNA methylation in both cell lines. 

The MSssI cell line, which overexpresses a CpG-specific bacterial DNMT MSssI, shows an increased overall CpG methylation of 20%, which is a clear increase compared to the untransfected (WT) and MCviPI mutant cell lines (Figure 1A). Moreover, the MSssI cell line shows almost no GpC methylation, indicating that the MSssI DNMT induces predominantly CpG methylation (Figure 1B). The MCviPI cell line overexpressing the viral GpC DNMT MCviPI shows an increased global GpC methylation of 20%, which is a clear increase compared to the control WT and MCviPI mutant cell lines (Figure 1B). Interestingly, the MCviPI cell line also reveals a global 10% increase in CpG methylation, which is not present in the MCviPI mutant cell line (Figure 1A). This suggests that the MCviPI GpC-inducing DNMT also elicits partial CpG methylation in the mitochondrial genome. As such, the MCviPI mutant overexpressing cell line acts as a robust negative control for both CpG and GpC mtDNA methylation. Since the amount of CpG methylation in the MCviPI cell line is only half the methylation increase in the MSssI cell line, there is a clear difference in mitochondrial methylation patterns between the MSssI and MCviPI cell lines, allowing us to compare the relative contribution of CpG and GpC methylation to mitochondrial regulatory functions.

### 2.2. MtDNA GpC Hypermethylation Promotes Specific Changes in Bile Acid Metabolic Gene Expression

Since there is a clear increase in mitochondrial CpG and GpC mtDNA methylation in both overexpressing cell lines (MSssI and MCviPI) as compared to the MCviPI mutant cell line, we next evaluated whether this differential mtDNA methylation also affects cellular (metabolic) gene expression. Since MCviPI mutant overexpressing and WT cells showed similar background CpG and GpC methylation levels (see Figure 1), in further experiments, we only included the MCviPI mutant overexpressing cell line as a reference (negative control) cell line. As such, all three cell lines received similar transfection-selection conditions. Mitochondrial localization of the overexpressed MSssI and MCviPI DNMTs in the HepG2 cells was confirmed by immunofluorescence microscopy, as previously shown in HCT116 and C33A cells [27] (Supplementary Figure S1). Moreover, in line with earlier observations of Van Der Wijst et al. [27], we did not detect major gene expression changes in selective mitochondrially encoded genes (Supplementary Figure S2). However, RNA sequencing identified 43 and 650 uniquely differentially expressed nuclear genes (DEG) upon mitochondrial CpG or GpC methylation, respectively, without further treatment with FFAs (Supplementary Figure S3). The Venn diagram identifies 58 common genes, primarily involved in metabolic processes, that are differentially expressed in both the MSssI (CpG) and MCviPI (GpC) DNMT-overexpressing cell lines (Supplementary Figure S3, Supplementary Table S2). 

**Figure 2:**
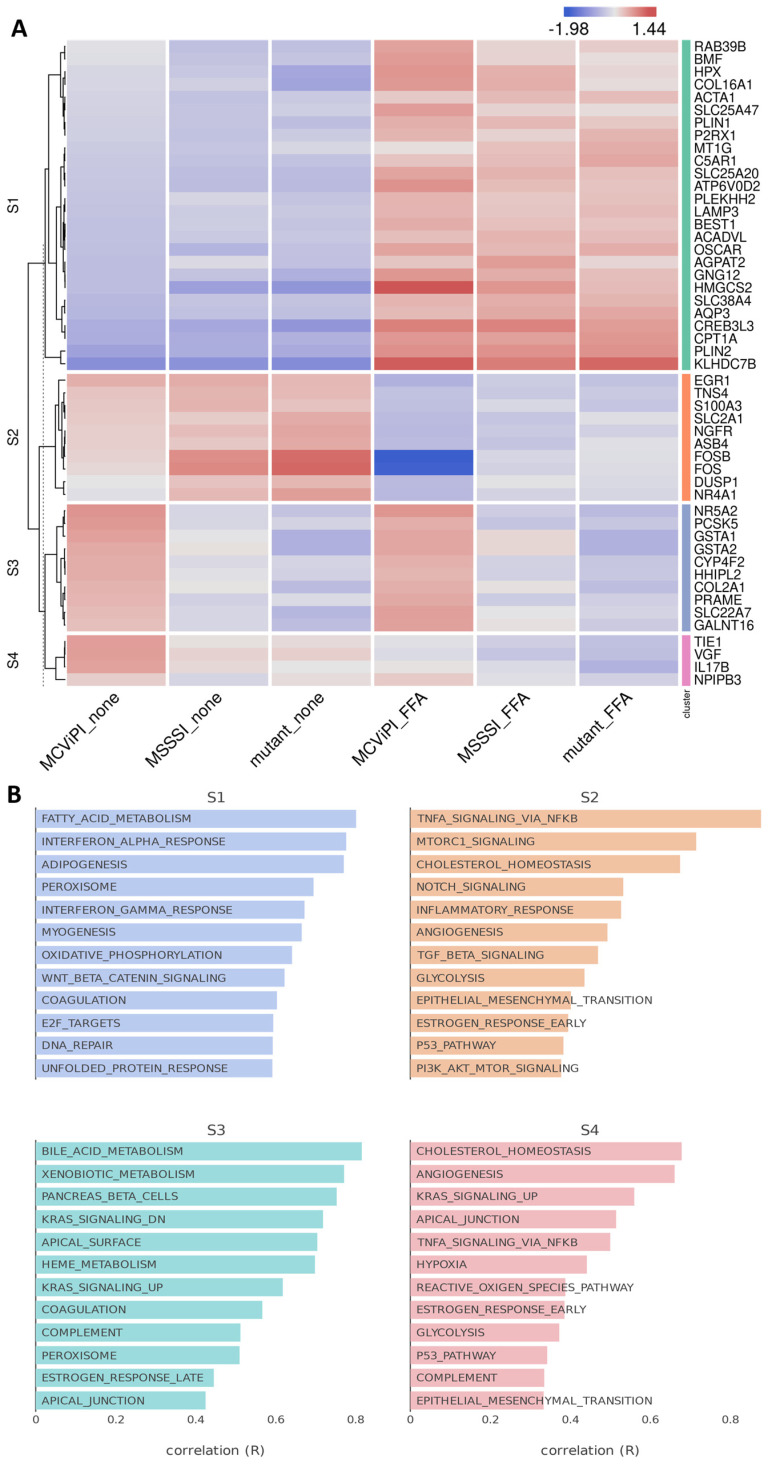
Mitochondrial GpC hypermethylation specifically upregulates bile acid metabolism. (**A**) Heatmap representation of differentially expressed genes in three different HepG2 cell lines overexpressing a GpC DNMT MCviPI (MCviPI), a CpG DNMT (MSSSI), or overexpressing a GpC/CpG DNMT deficient MCviPI mutant. Cells were left untreated (none) or treated with 1 mM FFA for 24 h (FFA) to induce a MASLD phenotype in vitro. (**B**) Pathway enrichment analysis of four main differentially expressed gene clusters.

Next, integrated Bigomics^TM^-based pathway enrichment analysis of the DEG revealed that FFA treatment induces a similar upregulation of fatty acid metabolism (gene cluster S1) and downregulation of genes involved in TNF/mTORC signaling and cholesterol homeostasis (gene cluster S2) in all three cell lines, irrespective of the mtDNA methylation status (Figure 2A,B). Interestingly, MCviPI overexpressing cells and, to a much lesser extent, MSssI overexpressing cells promote specific upregulation of genes involved in bile acid metabolism (gene cluster 3) (Figure 2A,B). The latter suggests that GpC rather than CpG mtDNA methylation elicits changes in bile acid metabolic gene expression. This differential gene expression by mtDNA methylation was further validated with qPCR of four genes related to bile acid metabolism or stress pathways: *GSTA1*, *GSTA2*, *SLC22A7*, and *NR5A2*, showing results in line with the RNAseq data (Supplementary Figure S4). 

**Figure 3:**
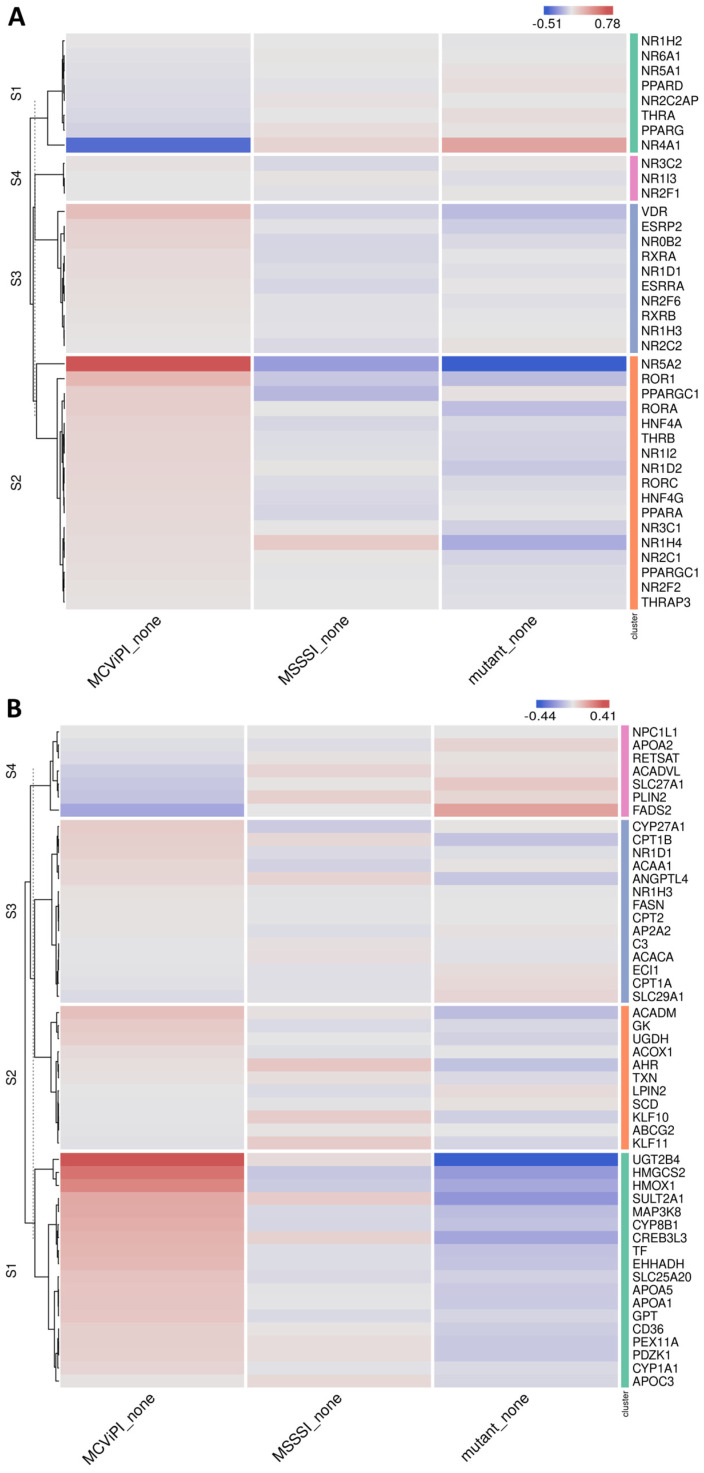
Mitochondrial GpC hypermethylation upregulates nuclear receptors and PPARα target genes involved in bile acid metabolism. Heatmap representation of differentially expressed nuclear receptors (**A**) or PPARα target genes (**B**) in three different untreated (none) HepG2 cell lines overexpressing a GpC DNMT MCviPI (MCviPI), a CpG DNMT (MSSSI), or overexpressing a GpC/CpG DNMT deficient MCviPI mutant.

**Figure 4:**
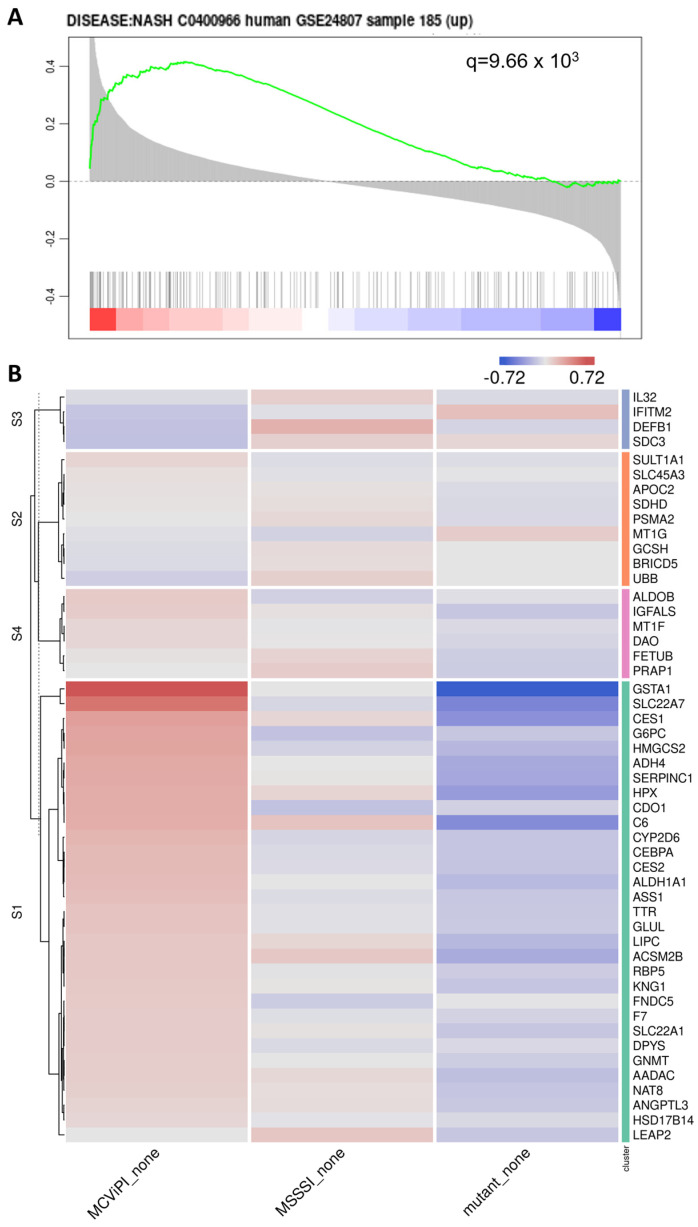
Mitochondrial GpC hypermethylation gene expression changes show enrichment of a MASLD gene signature. (**A**) Functional GSEA enrichment of MASLD-associated genes correlating with mitochondrial GpC methylation (MCviPI_none vs. Mutant_none). The green curve corresponds to the ‘running statistics’ of the enrichment score (ES). The more the green ES curve is shifted to the upper left of the graph, the more the gene set is enriched in the first group. Black vertical bars indicate the rank of genes in the gene set in the sorted correlation metric. FDR is represented by the q-value in the figure. The figure was generated using the Omics Playground tool (v3). (**B**) Heatmap representation of MASH-related gene signature (GSE24807) in 3 different HepG2 cells overexpressing a GpC DNMT MCviPI (MCviPI), a CpG DNMT (MSSSI), or overexpressing a GpC/CpG DNMT deficient MCviPI mutant. Cells were left untreated (none).

Of special interest, upon further searching for corresponding changes in nuclear hormone receptors involved in metabolic gene expression (Figure 3A), we identified increased expression of various key nuclear receptors involved in bile acid/fatty acid metabolism (i.e., *NR5A2*, *NR0B2*, *NR1H3*, *NR1I2*, *NR1H4*, *PPARα*, *HNF4* [28]), as well as elevated expression of multiple PPARα target genes (*CYP8B1*, *UGT2B4*, *SULT2A1*) involved in bile acid metabolism [29] in the MCviPI cell line (Figure 3B). Furthermore, protein–protein interaction analysis (https://string-db.org/, accessed on 23 August 2023) shows a strong interaction of the GpC mtDNA methylation-specific gene expression changes with key regulatory proteins for bile acid metabolism (Supplementary Figure S5). Interestingly, MCviPI mtDNA methylation-specific gene expression changes also directly reveal an enrichment of a human MASLD/MASH gene signature (Figure 4A,B).

### 2.3. MtDNA GpC/CpG Hypermethylation Modulates Mito-Nuclear Epigenetic Crosstalk

**Figure 5:**
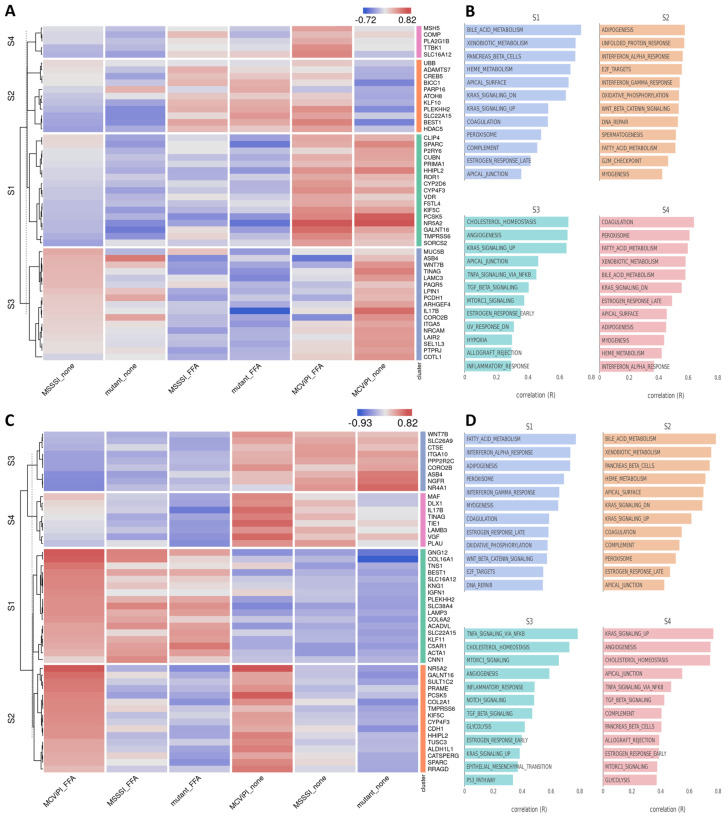
Mitochondrial GpC hypermethylation changes bile acid metabolism through epigenetic mito-nuclear communication. Heatmap representation of four differentially expressed gene clusters that are also differently methylated (DB > |0.1|) in MCviPI versus McviPI mutant cells left untreated (**A**,**B**) or treated with 1 mM FFA (**C**,**D**).

The concept of “mito-nuclear communication” refers to the interplay of mitochondrial dynamics with nuclear epigenetic regulation to adapt to environmental metabolic (energetic) challenges. Since the mitochondria regulate the production of the universal methyl donor s-adenosyl methionine (SAM) by the production of ATP and folate, this may also affect both mitochondrial as well as nuclear epigenetics [30,31]. Since our RNA sequencing results revealed predominant mitochondrial GpC hypermethylation-induced changes in bile acid metabolic gene expression, we next characterized whether this may also translate into crosstalk with nuclear epigenetic DNA methylation changes in the MCviPI overexpressing cell line. Upon analysis of the Illumina Epic 850K bead array β-value DNA methylation signal intensities in MCviPI and MCviPI mutant overexpressing HepG2 cell lines left untreated or treated for 24 h with FFA, we could identify 1754 DMPs in the untreated or 7565 DMPs in the FFA-treated MCviPI cell line as compared to the mutant counterpart cell line (DB > |0.1|). Upon cross-comparing differentially methylated genes (DB > |0.1|) with lists of differentially expressed genes derived from the RNAseq data via the integrative Bigomics^TM^ platform, we identified various nuclear epigenetic-controlled gene clusters that are enriched in processes related to fatty acid metabolism, cholesterol metabolism, bile acid metabolism, unfolded protein response, or TNF signaling (Figure 5). Of special note, differential methylation of various genes involved in bile acid metabolism can only exclusively be detected in MCviPI GpC mtDNA cells but not in the MCviPI mutant cell line, irrespective of the treatment. Altogether, these results strongly support the concept of mito-nuclear epigenetic communication between both compartments to regulate bile acid metabolism.

### 2.4. MtDNA GpC/CpG Hypermethylation Promotes Functional Mitochondrial Changes in Respiration and Morphological Features Associated with Mitophagy Stress Response

The preceding findings indicated significant alterations in gene expression patterns within metabolic pathways, closely linked to mitochondrial functioning. Therefore, in the subsequent experiments, we integrated various experimental approaches to assess potential functional changes in mitochondrial morphology, respiration, and ROS-lipid peroxidation damage associated with mtDNA GpC/CpG hypermethylation in MCviPI and MSssI cell lines versus deficient DNMT MCviPI mutant cells.

**Figure 6:**
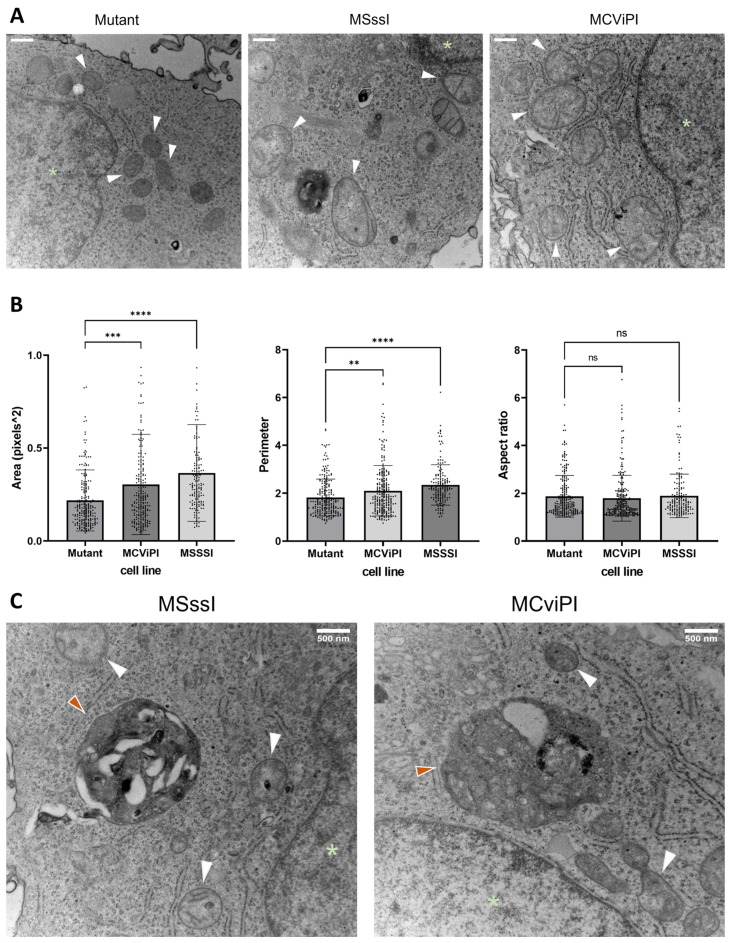
Mitochondrial hypermethylation-induced morphological changes. (**A**) Representative images of TEM of the reference MCviPI mutant cell line and the mitochondrially CpG or GpC methylated cell lines MSssI and MCviPI, respectively. White arrowheads indicate mitochondria, and green asterisks indicate the nucleus (scale bar (upper left) represents 500 nm). (**B**) quantification of surface area, perimeter, and aspect ratio of the mitochondria in the three cell lines (n = 2 biologically independent samples). Each data point represents a different mitochondrium (n = 158–234 mitochondria). Data are shown as mean ± s.d.; (ns = not significant; ** *p* < 0.01, *** *p* < 0.001, **** *p* < 0.0001, One-way ANOVA with Dunnett’s correction for multiple comparisons). (**C**) Representative images of TEM imaging of the mitochondrially CpG or GpC-methylated cell lines MSssI and MCviPI, respectively. White arrowheads indicate mitochondria, a green asterisk indicates the nucleus, and orange arrowheads indicate autophagosomes.

First, we applied electron microscopy to compare mitochondrial morphology in the MSssI and MCviPI overexpressing cell lines compared to the MCviPI mutant reference cell line. Therefore, we quantified three aspects of the mitochondrial shape, including the area, aspect ratio, and perimeter, indicating the size (area and perimeter) and shape (aspect ratio) of the mitochondria. Both the area and perimeter, representing the size, are significantly increased in the MSssI and MCviPI overexpressing cell lines as compared to the MCviPI mutant reference cell line, indicating that GpC/CpG mtDNA hypermethylation is associated with mitochondrial swelling (Figure 6B). However, the aspect ratio is not increased compared to the MCviPI mutant cell line because the overall shape of the mitochondria is not changed. These morphological changes are also clear from the images of the mitochondria showing overall mitochondrial swelling and disruption of the cristae structure in the MSssI and MCviPI cell lines, which is less prominent in the MCviPI mutant cell line (Figure 6A). Furthermore, autophagosomes could be detected in the MSssI and MCviPI cell lines, which were less frequently observed in the MCviPI mutant cell line, suggesting possible involvement of mitophagy-autophagy mitochondrial stress responses following GpC/CpG mtDNA hypermethylation (Figure 6C).

**Figure 7:**
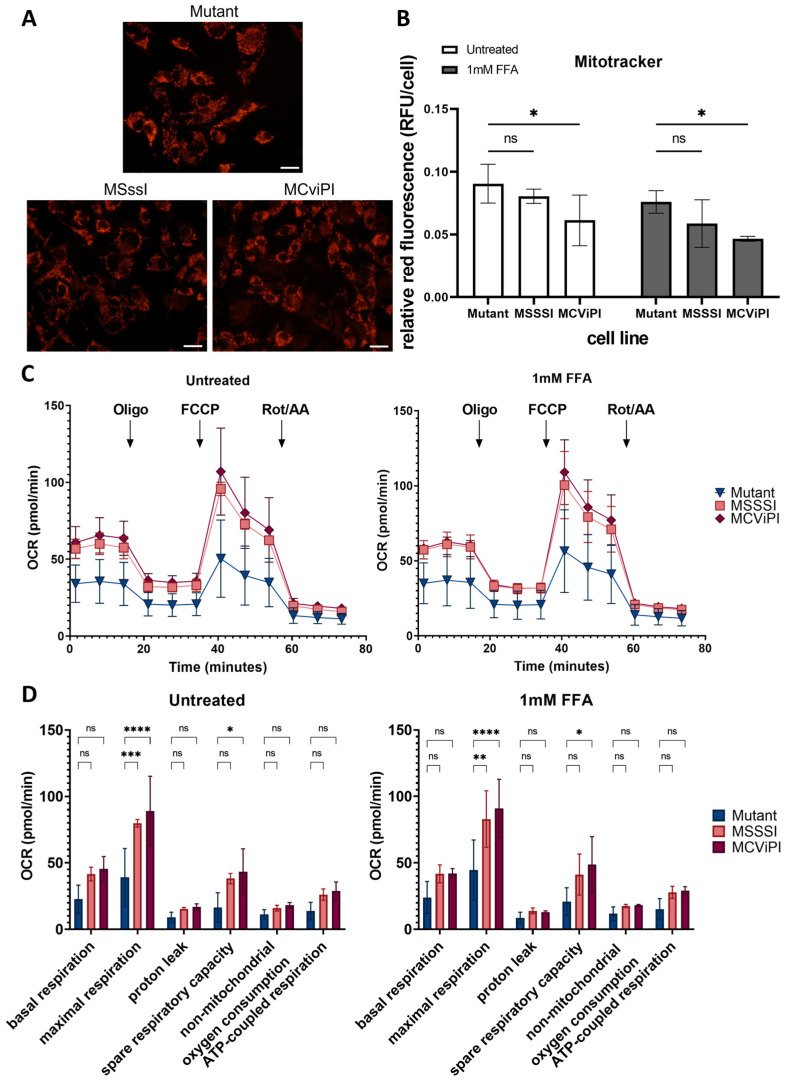
Characterization of mitochondrial distribution and metabolic function upon mitochondrial CpG/GpC hypermethylation. (**A**) Mitochondria stained with Mitotracker Red CMXRos dye in untreated cells (scale bar represents 100µM), and (**B**) Quantification showing relative fluorescence per cell based on the overall fluorescence to the total amount of cells in all cell lines (MCviPI mutant (mutant), MsssI (MSSSI), McviPI, and WT) (n = 3 independent biological replicates, two-way ANOVA test with Šidák’s correction for multiple comparisons). (**C**) The Seahorse XF Cell Mito Stress assay was used to measure changes in oxygen consumption rate after different triggers that inhibit or activate mitochondrial respiration (Oligo = oligomycin; FFCP; Rot/AA = rotenone and antimycin), both untreated (left) and treated with 1 mM FFA for 24 h cells (right). (**D**) Quantification of several aspects of mitochondrial respiration based on changes in oxygen consumption. Data are shown as mean ± s.d.; n = 3 independent biological replicates (ns = not significant; * *p* < 0.05, ** *p* < 0.01, *** *p* < 0.001, **** *p* < 0.0001, two-way ANOVA with Tukey’s correction for multiple comparisons).

Since the results of the TEM show morphological abnormalities caused by mtDNA GpC/CpG hypermethylation, we next compared possible effects on the cellular distribution and localization of mitochondria. Therefore, mitochondria were stained with Mitotracker Red CMXRos dye, which accumulates in active mitochondria in a potential-dependent manner, allowing us to visualize and study their distribution (Figure 7A). Our results show that GpC/CpG mtDNA hypermethylation in both MCviPI and MSssI cells does not influence the localization of the mitochondria. Mitochondria could be observed equally distributed all over the cytoplasm around the nucleus in all cell lines. Furthermore, when examining fluorescence intensity, both the MCviPI and MSssI cell lines display a slight decrease compared to the mutant MCviPI cell line, whether they were left untreated (0.061 ± 0.020 and 0.080 ± 0.006 vs. 0.090 ± 0.016) or treated with 1 mM FFA (0.046 ± 0.002 and 0.059 ± 0.019 vs. 0.076 ± 0.009). However, this decrease reached only statistical significance in the MCviPI cell line (Figure 7B). 

Taking into account the observed morphological and phenotypic mitochondrial alterations, we next performed a mitochondrial stress test using Agilent Seahorse XF Technology. This assay allows us to assess different aspects of mitochondrial respiration, such as basal respiration, ATP-coupled respiration, and maximal respiration, based on differences in oxygen consumption rate (OCR) upon GpC/CpG mtDNA hypermethylation. MSssI, MCviPI, and MCviPI mutant overexpressing cell lines were either left untreated or treated 24 h with 1 mM FFA to simulate a steatosis phenotype in vitro, allowing us to assess the corresponding mitochondrial respiration. Figure 7C,D shows that MSssI and MCviPI DNMT overexpressing cell lines have increased overall respiration, both treated and untreated, compared to the GpC/CpG-deficient MCviPI mutant cell line. This is reflected by a significant increase in maximal respiration for both cell lines. In addition, the spare respiratory capacity is also increased in both cell lines, although not statistically significant in the MSssI cell line (Figure 7D). These results suggest that GpC and CpG mtDNA hypermethylation promote increased metabolic activity in the mitochondria. However, this increased respiratory capacity cannot be further enhanced in the presence of 1 mM FFA for 24 h.

**Figure 8:**
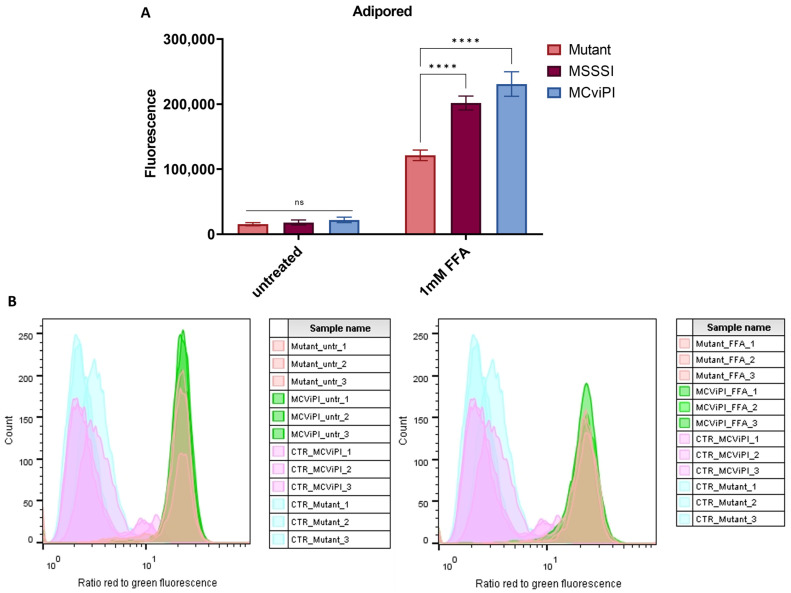
Mitochondrial hypermethylation induces lipid accumulation under lipid stress, without further lipid peroxidation. (**A**) Quantification of lipid droplets in both untreated and 24 h treated with 1 mM FFA cell lines (MCviPI mutant (mutant), MsssI (MSSSI), and MCviPI) with Adipored fluorescent staining. Data are shown as mean ± s.d.; n = 3 independent biological replicates (ns = not significant; **** *p* < 0.0001, two-way ANOVA with Tukey’s correction for multiple comparisons). (**B**) Lipid peroxidation quantification with the Image-iT Lipid Peroxidation kit using flow cytometry. The lipid peroxidation reagent is a ratiometric probe, and the signal is detected on a flow cytometer with 488 nm laser excitation and fluorescence emission measured at 530/30 nm and 532 nm laser excitation and fluorescence emission measured at 585/42 nm. The data are represented as the ratio of red/green fluorescence intensities. Ratios are lower (indicating greener signal) in cells treated with cumene hydroperoxide (positive control; CTR), but there is no difference between ratios of the MCviPI mutant (Mutant) and MCviPI cell line, both untreated and treated with 1 mM FFA for 24 h (n = 3 independent technical replicates).

Since byproducts of aerobic respiration in the mitochondria are free radicals (ROS), which frequently trigger lipid peroxidation damage, we next compared levels of lipid peroxidation ROS damage in the different cell lines left untreated or upon lipid accumulation following treatment with 1 mM FFA. First, lipid accumulation was quantified with an Adipored staining of lipid droplets, showing a clear accumulation of lipid droplets by treatment with 1 mM FFA in all cell lines, irrespective of the mtDNA methylation status. Moreover, a significant increase in lipid droplets was found in the MSssI/MCviPI DNMT overexpressing cell lines compared to the reference MCviPI mutant cell line (Figure 8A). Next, ROS lipid peroxidation damage was quantified via flow cytometry using a fluorescent BODIPY™ 581/591 C11 reagent. This reagent localizes to membranes throughout live cells and, upon oxidation by lipid hydroperoxides, displays a shift in peak fluorescence emission from ~590 nm to ~510 nm, providing a ratiometric indication of lipid peroxidation levels. However, no significant difference in lipid peroxidation levels could be observed between MCviPI or MCviPI mutant cell lines left untreated or in FFA-treated conditions (Figure 8B). Along the same line, a lipid peroxidation-dependent cell death assay revealed no significant changes in sensitivity to ferroptosis upon treatment with the ferroptosis inducer compound RSL3 (Supplementary Figure S6).

### 2.5. MtDNA GpC-CpG Hypermethylation Promotes Cholestasis-Associated Autophagy-Mitophagy Stress Response

Based on the MCviPI GpC mtDNA hypermethylation-associated gene expression changes in bile acid metabolism (Figure 2), as well as mitochondrial morphology changes (Figure 6A) and increased respiratory functions (Figure 7C), we next performed a more specialized GSEA enrichment analysis of cellular and mitochondrial stress gene signatures [32,33,34]. 

**Figure 9:**
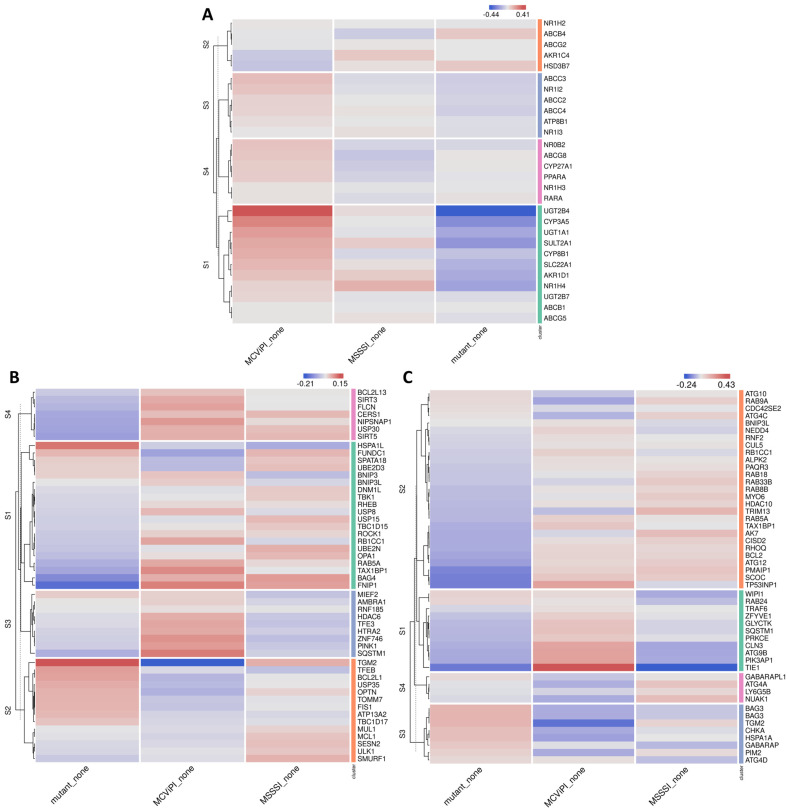
Mitochondrial CpG and GpC hypermethylation induce upregulation of mitophagy- and autophagy-related genes. Heatmap representation of differentially expressed genes related to (**A**) cholestasis, (**B**) mitophagy, and (**C**) autophagy in 3 different untreated cell lines, including a cell line overexpressing an inactive MCviPI DNMT named MCviPI mutant (Mutant) and the MCviPI cell line or MSssI cell line overexpressing the GpC DNMT MCviPI and CpG DNMT MSssI, respectively. (n = 3 biologically independent replicates).

First, we checked the cholestasis signature, which is typically related to increased bile acid metabolism. Interestingly, the MCviPI overexpressing cell line (MCviPI) shows a significant enrichment of the cholestasis disease signature compared to the MSssI and MCviPI mutant overexpressing cell lines. The mechanisms by which cholestasis induces liver damage due to the accumulation of bile acids include mitochondrial dysfunction, oxidative stress, and ER stress. These cellular stressors typically further induce cell death and stimulate an integrated autophagy-mitophagy (also known as “cholestophagy”) stress response as a compensatory mechanism aiming to reduce (liver) damage [35]. Accordingly, besides cholestasis, we also identified the most prominent upregulation of autophagy and mitophagy pathways in the MCviPI and MSssI overexpressing cell lines as compared to the MCviPI mutant cell line (Figure 9).

Together, these results suggest that hypermethylated mitochondria are sensed as functionally overactivated damaged mitochondria, which need to be cleared from the cell to limit liver toxicity, aiming to restore metabolic homeostasis. Interestingly, this is highly similar to the mitochondrial dysfunction in the progression from steatosis to steatohepatitis and fibrosis in MASLD [35,36]. 

## 3. Discussion

In follow-up of a pilot study by Mposhi et al. [21] showing first evidence for the impact of targeted mitochondrial CpG (MSssI) and GpC (MCviPI) DNA methylation on MASH-associated gene expression, we here further characterized mito-nuclear epigenetic crosstalk by functional mitochondrial changes in morphology, respiratory activity, and metabolic competence during MASLD lipid stress in relation to gene expression and (mito)epigenetic signatures. 

First, nanopore episequencing of the mtDNA of the different cell lines overexpressing a MCviPI (GpC), MSssI (CpG), or a MCviPI mutant-deficient DNMT targeted to the mitochondria confirmed increased mitochondrial GpC and CpG methylation in the MCviPI and MSssI cell lines, respectively. Importantly, the observed augmentation in CpG and GpC methylation was absent in the MCviPI mutant cell line, showing a similar methylation pattern to the untransfected HepG2 cells. Remarkably, besides GpC methylation, MCviPI overexpressing cells also showed increased CpG methylation levels, although weaker than the CpG methylation levels achieved in the MSssI overexpressing cell line. This suggests the possibility of dual GpC/CpG mtDNA methylation properties of the MCviPI DNMT. Overall, low mtDNA methylation levels were observed in the MCviPI mutant cell line, similar to untransfected HepG2 cells. The average 4% CpG methylation found in both cell lines is similar to the 3.4% methylation found by Lüth et al. in healthy controls upon Nanopolish analysis of the Nanopore data [37]. In addition, similar methylation levels were reported by Goldsmith et al. in HepaRG cells [38]. Of special note, the research of Goldsmith et al. also showed that higher percentages of mtDNA methylation can be found in tissue as compared to cell lines. Interestingly, the untransfected and MCviPI mutant HepG2 cell lines show higher baseline CpG than GpC methylation levels, which suggests that mitochondrial DNA is more susceptible to CpG methylation than GpC methylation. Similarly, Goldsmith et al. observed more CpG methylation than non-CpG methylation in liver tissue [38]. Nevertheless, strand-specific GpC methylation in the D-loop of human mtDNA samples has previously shown associations with mitochondrial transcription changes and therefore remains important to study [39,40]. In our DNMT-overexpressing cell lines, an average CpG/GpC mtDNA methylation of 20% could be detected. While this approach gives the opportunity to directly investigate the functional effects of mitochondrial CpG/GpC DNA hypermethylation, it is important to acknowledge that the artificially achieved percentages resulting from mitochondrial DNMT overexpression surpass the physiologically observed DNA methylation levels. In control and patient tissue, CpG methylation levels ranged from 5.12 to 5.96%, while non-CpG methylation levels were observed in the range of 0.12 to 0.15% [38]. 

Hypermethylation of nuclear genes is generally associated with gene repression. Interestingly, in line with Van Der Wijst et al., we found that essentially only mitochondrial GpC methylation induces a minor downregulation of selective mitochondrial genes (Supplementary Figure S2) [27]. In addition, recent papers show that, besides correct transcription, mitochondrial protein translation and therefore mitochondrial function are also dependent on correct post-transcriptional RNA modifications [41,42]. Furthermore, we found that mitochondrial GpC and, to a lesser extent, CpG methylation specifically increase nuclear bile acid metabolic gene expression. Accordingly, expression of multiple key nuclear receptors involved in regulation of bile acid/fatty acid/cholesterol/lipid metabolism (i.e., *NR5A2* (*LRH1*), *NR0B2* (*SHP*), *NR1H3* (*LXRa*), *NR1I2* (*PXR*/*SXR*), *NR1H4* (*FXR*), *PPARα*, *HNF4*) [28,43] was increased, which resulted in elevated expression of various PPARα target genes (*CYP8B1*, *UGT2B4*, *SULT2A1*) involved in bile acid metabolism. Moreover, similar to Schiöth et al., we observed multiple nuclear DNA methylation changes in various bile acid metabolic genes upon MASLD-related lipid stress [44]. Interestingly, the methylation changes in bile acid metabolic genes in our data were specifically associated with GpC mtDNA hypermethylation, irrespective of FFA treatment, which confirms important mito-nuclear metabolic crosstalk in epigenetic regulation and gene expression [45]. 

Hepatic bile acid synthesis is the major catabolic mechanism for cholesterol elimination from the body and is strictly regulated. Interestingly, recent studies identified bile acid metabolic dysregulation-induced cholestasis as a key factor in steatohepatitis disease etiology in MASLD [46,47,48,49,50,51]. In line, we also found enrichment of MASH (Figure 4) and cholestasis disease signatures (Figure 9A) associated with the epigenetic remodeled increased bile acid metabolic gene expression network in MCviPI overexpressing cells with GpC mtDNA hypermethylation. 

Furthermore, new evidence links disturbed bile acid metabolism to mitochondrial dysfunctions, including changes in respiration, mitochondrial swelling, and a decrease in mitochondrial transmembrane potential [52,53,54]. Interestingly, similar mitochondrial malfunctions also contribute to MASLD/MASH disease etiology [55], implicating a contribution of bile acid dysfunction in the mitochondrial dysfunction of MASLD/MASH patients. As a compensation mechanism to cope with bile acid metabolic stress and avoid liver injury, bile acids promote selective hepatic “cholestophagy” to get rid of cholestasis-induced damage and thereby maintain cellular integrity and energy homeostasis. This process involves a complex interplay of autophagy–mitophagy–lipophagy pathways, regulated by bile acids and the bile acid receptor NR1H4 (FXR) [35,36,56]. In line, electron microscopy experiments showed a mix of regular and aberrant-shaped mitochondria, revealing more mitochondrial swelling and autophagosome formation in MCviPI, as well as MSssI overexpressing cells with GpC/CpG mtDNA hypermethylation, but absent in MCviPI mutant methylation deficient cells (Figure 6A,B). This may reflect a higher mitochondrial turnover rate in cells with GpC/CpG mtDNA hypermethylation to remove “dysfunctional” mitochondria by mitophagy-autophagy [57]. In line with this hypothesis, our RNA sequencing also revealed increased expression of multiple genes related to mitophagy and autophagy pathways in the MCviPI and MSssI overexpressing cell lines. Interestingly, the MCviPI and MSssI cell lines do not always upregulate the same genes, suggesting different degrees or types of metabolic stress induced by mitochondrial GpC or CpG methylation that activate different mitophagy and autophagy regulation. 

Altogether, these findings suggest that mitochondrial GpC/CpG methylation elicits metabolic stress damage, which needs to be overcome by a fast turnover of dysfunctional mitochondria through the process of mitophagy. Consequently, mitochondrial methylation could represent another risk factor in MASLD, given the mounting body of evidence demonstrating a robust correlation between impaired mitophagy–autophagy and the progression of MASLD [58,59,60,61]. 

Further characterization of the metabolic changes induced by mtDNA CpG/GpC methylation showed that, under in vitro steatotic conditions following FFA treatment, MCviPI and MSssI overexpressing cells with GpC/CpG mtDNA hypermethylation show a similar increase in free fatty acid metabolic gene expression as MCviPI mutant mtDNA methylation deficient cells (Gene cluster S1 Figure 2). However, in contrast, mitochondrial respiration is only significantly increased in MCviPI and MSssI overexpressing cells with GpC/CpG mtDNA hypermethylation as compared to MCviPI mutant cells (Figure 7C,D). This divergence in respiratory activity might be attributed to the upregulation of additional nuclear receptors (i.e., *NR5a2* (*LRH1*)), known to be involved in upregulation of mitochondrial respiration, as a direct consequence of GpC/CpG mtDNA hypermethylation [62] (Figure 3A). Although the comprehensive mechanism has not been resolved, it is noteworthy that the overactivated mitochondrial respiration observed in the methylated cell lines (MSssI and MCviPI) could not be further elevated following treatment with FFA. Intriguingly, this lack of response to FFA treatment coincided with the induction of lipid accumulation, as depicted in Figure 8A. Moreover, this maximal hyperactivation of the mitochondria may slowly promote the decline (exhaustion) of mitochondrial activity, as was already slightly observed by the decreasing fluorescent intensities by Mitotracker staining (Figure 7A,B). Interestingly, increased basal mitochondrial respiration and maximum respiration have been observed as an adaptive mitochondrial response in early steatosis. However, this will eventually induce structural mitochondrial deformations and metabolic shutdown of mitochondrial metabolism in the MASH stage, which is similar to the mitochondrial methylation-induced metabolic changes in our results [23,59,63]. Surprisingly, despite increased levels of lipid accumulation and mitochondrial respiration activity in MCviPI and MSssI overexpressing cells as compared to MCviPI mutant cells, we could not detect increased levels of lipid peroxidation damage or ferroptosis sensitivity. Remarkably, disturbed cholesterol homeostasis has recently been found to trigger ferroptosis resistance [64]. However, how bile acids and cholestasis specifically promote ferroptosis resistance needs further investigation. 

In summary, our study represents the first evidence of mitochondrial GpC methylation initiating a novel phenomenon termed “cholestasis-induced mitophagy” or “cholestophagy” through the alteration of mito-nuclear epigenetic alterations within the bile acid metabolism. Furthermore, both mitochondrial CpG and GpC methylation induce a basal state of mitochondrial overactivity, leading to lipid accumulation in response to lipid stress, accompanied by morphological changes that promote mitophagy. Consequently, future therapeutic investigations targeting mitochondrial DNA methylation present a promising avenue for mitigating the progression by reversing the epigenomic conditions that cause MASLD.

## 4. Materials and Methods

### 4.1. Cell Culture

The human hepatoma cells (HepG2) cell lines overexpressing mitochondria-targeted DNMTs MCviPI, MsssI, or inactivated MCviPI (MCviPI mutant) and the un-transfected control (WT) HepG2 cells were a kind gift of Prof. Dr. Marianne Rots (UMCG, Groningen, The Nertherlands) [21]. The overexpressing cell lines were constructed as described by Van der Wijst et al. [27]. Briefly, the sequence of the MCviPI, MCviPI mutant (catalytically inactive), and MSssI was cloned in a pCDH-CMV-MCS-SV40-puro plasmid, resulting in a pCDH-CMV-master synthetic construct-conII-SV40-puro containing a mitochondrial localization signal (MLS) followed by [MCviPI/MCviPI mutant/MSssI] and two nuclear export signals (NES). This construct was subsequently lentiviral transduced into HepG2 cells, followed by antibiotic selection with puromycin for positive clones. 

Cells were cultured in Dulbecco’s modified Eagle medium (DMEM, Gibco, Thermo Fisher Scientific, Dilbeek, Belgium, 41965039) supplemented with 10% fetal bovine serum (Gibco, Thermo Fisher Scientific, Dilbeek, Belgium, 10270106), 1% penicillin-streptomycin solution (Gibco, Thermo Fisher Scientific, Dilbeek, Belgium, 15140122), and 1 mM pyruvate. Both cell lines were cultured in T75 or T25 flasks in a humidified atmosphere (37 °C and 5% CO_2_).

### 4.2. FFA Medium

The FFAs medium to obtain MASLD-like conditions is composed of oleic (OA) and palmitic (PA) acid in a 2:1 ratio. Stock solutions of 0.66M oleic acid and (1.32 M) palmitic acid (Sigma-Aldrich, Taufkirchen, Germany) were prepared in isopropanol. Equal amounts of oleic and palmitic acid were mixed to prepare a stock of 1 mM FFA. FFA-free bovine serum albumin (BSA) was dissolved in serum-free DMEM medium without antibiotics at the final concentration of 1% and then sterilized using syringe-driven 0.22 μm filters. Afterwards, the medium was supplemented with the mixture of FFA at the final concentration of 1 mM and sonicated for 6–8 h until FFA was completely dissolved using a Branson 3200 sonication bath. The FFA medium was protected from light and stored at 4 °C.

### 4.3. Nanopore Sequencing

DNA was extracted using the Qiagen Blood Tissue DNA Isolation Kit (Qiagen, Antwerp, Belgium, 69504) according to the manufacturer’s protocol. The quality of the extracted DNA was measured using the Qubit 4 Fluorometer (Thermo Fisher Scientific, Q33238) and Qubit™ dsDNA BR kit (Thermofisher, Dilbeek, Belgium, Q32850) for concentration, Little Lunatic (Unchained Labs, Gent, belgium) for purity, and the Fragment Analyzer (DNF-492 Large Fragment Kit, Agilent, Diegem, Belgium) for integrity using either the “Agilent DNF-464 HS Large Fragment Kit” (integrity of extracted hmw-DNA) or the “Agilent DNF-492 Large Fragment Kit” (fragmentation and size selection). After quality control, 5 µg of DNA was fragmented using Megaruptor 3 (Diagenode) to a final fragment sizing 15–20 kb, which also resulted in the linearization of mtDNA and the exposure of fragments’ ends for end repair and adapter ligation. After fragmentation, small molecules were depleted using the Short Read Eliminator Kit (SRE XS, PacBio, London, UK), depleting short DNA fragments <10 kb progressively and DNA <4 kb almost completely. Library preparation was started with 175 fmol of size-selected DNA per sample (+/−2 µg of fragmented, size-selected DNA) and consisted of FFPE DNA end repair in combination with a preparation of the ends for adapter attachment, native barcode ligation, and sequencing adapter ligation with the use of the Native barcoding expansion 13–24 kit (Oxford Nanopore Technologies, Oxford, UK, EXP-NBD114) in conjunction with the Ligation sequencing kit (Oxford Nanopore Technologies, SQK-LSK109). Prior to the final sequencing adapter ligation, samples were pooled equimolarly for optimal read distribution. Sequencing was performed on the R9.4.1 PromethION Flow Cell, which had 8750 pores available for sequencing. In total, 50 fmol of the final library was loaded on the flow cell (~550 ng). Total sequencing time was 80 h on PromethION 24 (Oxford Nanopore Technologies), with a flush using DNase I before loading a fresh library at 24 and 48 h of sequencing. The sequencing run produced 12.49 M reads with an N50 of 17.39 kb, resulting in a total base output of 153.16 Gb and a total amount of data of 1.2 TB. MtDNA is well covered when performing shallow gDNA sequencing (60–80× vs. 1× for gDNA). Reads were basecalled using GUPPY (version 6.0.6). Further analysis was performed using a pipeline integrated into genomecomb [65]. Reads were aligned to the hg38 genome reference [66] using minimap2 [67], and the resulting sam file was sorted and converted to bam using samtools [68]. Structural variants were called using sniffles [69], cuteSV [70], and npinv [71]. The resulting variant sets of different cell lines were combined and annotated using genomecomb [65]. Nanopolish analysis (version 0.13.2) [72] was used for CpG and GpC methylation analyses on the mitochondrial genome without applying NUMT filtering, as NUMTs were shown to only have a marginal impact on methylation assessment [72,73]. Raw nanopore epi-sequencing data of transgenic HepG2 cell models with mitochondrial overexpression of CpG (MSSSI) or GpC (MCviPI)-specific DNA methyltransferases [21] have been deposited in the NCBI GEO database with accession number PRJNA95689.

### 4.4. RNA Extraction and RNA Sequencing

Total RNA was extracted with the RNeasy kit (Qiagen, 75162) from the 3 cell lines (MCviPI mutant, MSssI, and MCviPI), both untreated or treated with 1 mM FFA for 24 h, according to the manufacturer’s protocol (n = 4 biological replicates per cell line per treatment, except untreated MCviPI mutant n = 3). Afterwards, RNA quantity was determined using the Qubit^TM^ RNA Broad Range Assay Kit (Thermo Fisher Scientific, Q10210) with the aid of the Invitrogen Qubit^TM^ Fluorometer (Thermo Fisher Scientific, Q33238). The extracted RNA was stored at −80 °C and subsequently sent to Novogene Leading Edge Genomic Services & Solutions, where RNA integrity was determined using the 2100 Bioanalyzer system (Agilent Technologies, Diegem, Belgium). All 40 samples with acceptable quality levels (RNA content ≥ 20 ng/μL, OD260/280 ≥ 2.0, and RIN ≥ 4.0) were included for sequencing library preparation and RNA sequencing analysis. In brief, messenger RNA was purified from total RNA using poly-T oligo-attached magnetic beads. After fragmentation, the first strand of cDNA was synthesized using random hexamer primers, followed by the second strand of cDNA synthesis and library construction. The library was checked with Qubit and real-time PCR for quantification and a bioanalyzer for size distribution detection. Quantified libraries were pooled and 150 bp paired-end sequenced on the Illumina Novaseq6000 platform. The quality of the raw sequencing reads was evaluated using FastQC (v0.11.5) [74], and subsequent alignment to the genome reference consortium human build 38 (GRCh38/hg38) was performed with the STAR (v.2.7.3a) tool [75]. Differential gene expression and pathway analysis were performed using DESeq2 R package software (v3.18) [76] and the Omics Playground tool (v2.8.12) platform, which was also used for further visualization. Protein interaction networks were generated using the STRING database (v11) [77]. RNA sequencing was validated by qPCR and deposited in the NCBI GEO database with accession number GSE241526.

### 4.5. Quantitative Polymerase Chain Reaction (qPCR)

After RNA extraction, total RNA was converted into cDNA with the iScriptTM cDNA Synthesis Kit (BioRad, Temse, Belgium, 1708890) according to the manufacturer’s protocol. Next, qPCR analysis was performed using the PowerUp SYBR^TM^ green PCR master mix (Thermo Fisher Scientific, Waltham, MA, USA) according to the manufacturer’s instructions. In brief, a 20 µL reaction volume mix per sample was prepared containing 10 µL PowerUp SYBR Green Master Mix, 0.4 µM forward and reverse primer, and nuclease-free water. The following PCR program was applied on the Rotor-Gene Q qPCR machine of Qiagen: 95 °C for 10 min, 40 cycli denaturation (95 °C, 15 s), annealing/extension (60 °C, 1 min), and dissociation (60–95 °C). Each sample was run in triplicate. The median value of the triplicates was taken to calculate the ΔΔCt-values using B2M as the normalization gene. B2M, GSTA1, GSTA2, NR5a2, SLC22a7, mtND1, mtCOX1, and mtCYB primer sequences (Supplementary Table S1) were designed by Primer3 and synthesized by Integrated DNA Technologies (IDT, Leuven, Belgium). Statistical analysis was carried out using a one-way ANOVA test with Tukey’s correction for multiple comparisons. A *p*-value < 0.05 was considered statistically significant.

### 4.6. Methylation Analysis

Whole-genome methylation profiling targeting over 935,000 CpG sites was performed on the total DNA of the MCviPI mutant and MCviPI using the Infinium MethylationEPIC array (Illumina, San Diego, CA, USA) at the Centre for Medical Genetics (UZA, University of Antwerp), both untreated or treated with 1 mM FFA for 24 h. Genomic DNA (gDNA) was extracted from the cells using the Dneasy Blood & Tissue Kit (Qiagen, 69504) according to the manufacturer’s protocol. DNA concentration and purity were determined by the Qubit 4 Fluorometer (Thermo Fisher Scientific, Q33238). Next, 750 ng of DNA was bisulphite converted with the EZ DNA Methylation Kit (Zymo Research, D5001/D5002, Irvine, CA, USA) according to the manufacturer’s instructions. Successful bisulphite conversion was confirmed by PCR with the PyroMark PCR kit (Qiagen) in a region of the Sall3 gene (Supplementary Table S1). The resulting PCR products were run on a 2% agarose gel. This converted DNA was then further hybridized with the Infinium MethylationEPIC array (Illumina, San Diego, CA, USA) according to the manufacturer’s instructions. In brief, converted DNA was amplified overnight and fragmented enzymatically. Subsequently, DNA was precipitated and resuspended in hybridization buffer and afterwards dispended onto the BeadChips. The hybridization procedure was performed at 48 °C overnight using an Illumina Hybridization oven. After hybridization, free DNA was washed away, and single nucleotide extension followed by fluorescent readout was performed. The BeadChips were imaged using an Illumina HiScan (Illumina, San Diego, CA, USA). The platform interrogates more than 935,000 methylation sites per sample at single-nucleotide resolution. Annotations for the interrogated sites were taken from Illumina’s BeadChip array manifest based on genome reference consortium human build 37 (GRCh37/hg19). Raw intensity data from IDAT files was read and processed in R (v. 4.2.0) via the minfi [78] R package. Data pre-processing consisted of masking probes with poor design, control probes, X-/Y chromosome probes, and non-cg and non-ch probes. Probes with detection *p*-values > 0.01 in more than 50% of the samples were filtered out. No samples had more than 10% missing values; thus, all were considered for further analysis. For quality control, the ratio of log2 median intensities (methylated and unmethylated) along with β-value densities was calculated. β-values were then further processed using ChAMP (v 2.21.1) [79]. The difference in signal intensity between the two-color channels (dye bias correction) was corrected using the beta mixture interquartile matrix (BMIQ) method [80]. Methylation levels were reported as β-values ranging from 0 for unmethylated probes to 1 for fully methylated probes. To identify significantly differentially methylated CpGs between the different groups, parametric linear mixed models were used via ChAMP [79]. *p*-values were adjusted for multiple testing using the Benjamini–Hochberg correction (*p* < 0.01). Further Metascape pathway analysis of genes with a delta beta (DB) > |0.1| and FDR < 0.05 was performed with the online Metascape Web tool [81]. Methylation data were deposited in the NCBI GEO database with accession number GSE240988.

### 4.7. Lipid Quantification with Adipored

The AdipoRed Adipogenesis Assay (Lonza, Walkersville, MD, USA) was used to quantify intracellular lipid accumulation in all 3 cell lines (MCviPI mutant, MSssI, and MCviPI), both untreated or treated with 1 mM FFA for 24 h according to the manufacturer’s protocol (n = 3 biologically independent samples per cell line per treatment). Briefly, the medium of treated cells was replaced by phosphate-buffered saline (PBS) and incubated with the AdipoRed reagent. Afterwards, fluorescence was measured at 485 nm excitation and 572 nm emission using a microplate reader (FLUOstar Omega, BMG Labtech, Ortenberg, Germany). Statistical analysis was carried out using a two-way ANOVA test with Tukey’s correction for multiple comparisons. A *p*-value < 0.05 was considered statistically significant.

### 4.8. Lipid Peroxidation

Cellular lipid reactive oxygen species were measured in live cells through oxidation of the BODIPYTM 581/591 C11 reagent using the Image-iT™ Lipid Peroxidation Kit (C10445, Thermo Fisher Scientific, Waltham, MA, USA) according to the manufacturer’s protocol. In short, cells were seeded in 6-well plates at a density of 5 × 10^4^ cells/well and treated the next day for 24 h with 1 mM FFA (2:1 ratio oleic acid and palmitic acid, respectively) or 2 h with 100 μM cumene hydroperoxide (positive control). Cells were subsequently incubated for 30 min with a 10μM Image-iT™ Lipid Peroxidation Sensor at 37 °C. After incubation, cells were collected by trypsinization with TrypLE Express Enzyme (Thermo Fisher Scientific, Waltham, MA, USA). Cells were washed three times with pre-warmed PBS, and the fluorescence shift from 590 nm to 510 nm, representing oxidation of the reagent by lipid hydroperoxides, was measured with the CytoFlex flow cytometer (Beckman Coulter Life Sciences, Indianapolis, IN, USA). Finally, the 510/590 ratio was calculated and visualized as a ratio showing a red-to-green shift. Hence, the more lipid peroxidation, the lower the red-to-green shift will be.

### 4.9. Mitotracker

MitoTracker^TM^ Red CMH2Xros (Thermofisher, M7513) was used to visualize and quantify mitochondria in all 3 cell lines (MCviPI mutant, MSssI, and MCviPI), both untreated or treated with 1 mM FFA for 24 h according to the manufacturer’s protocol (n = 3 biologically independent samples per cell line per treatment). Briefly, cells were seeded at a density of 4 × 10^4^ cells/well in a 96-well plate and treated with 1 mM FFA for 24 h the next day. Subsequently, medium was replaced with staining medium consisting of DMEM medium (DMEM, Gibco, Thermo Fisher Scientific, 41965039) without FBS with a final concentration of 250 nM Mitotracker and incubated for 30 min. Afterwards, medium was replaced by a complete medium, and cells were observed with the Olympus CKX53 fluorescence microscope (Olympus, Antwerp, Belgium), or red fluorescence was measured at 579 nm excitation and 599 nm emission using the Tecan Spark Cyto (Tecan, Männedorf, Switzerland). Statistical analysis was carried out using a two-way ANOVA test with Šidák’s correction for multiple comparisons. A *p*-value < 0.05 was considered statistically significant.

### 4.10. Immunofluorescence Staining

The three overexpressing HepG2 cell lines (MCviPI mutant, MSssI, and MCviPI) were seeded at a density of 0.3 × 10^6^ cells/well in a 6-well plate and incubated with 250 nm μM MitoTracker Red CMXRos (Invitrogen, Dilbeek, Belgium) for 30 min at 37 °C, 5% CO_2_. Cells were washed in PBS and fixed with 4% paraformaldehyde for 15 min, and then permeabilized and blocked with 0.1% Triton X-100 and 1% BSA in PBS for 1 h. Subsequently, cells were incubated with a primary antibody directed against HA-tag (Sigma-Aldrich, Taufkirchen, Germany H3663) diluted 1:250 in PBS containing 2.5% BSA overnight at 4 °C. After washing, they were incubated for 1 h at room temperature with goat anti-mouse Alexa Fluor™ 488 (Invitrogen; A-11001), diluted in 1:500 PBS containing 2.5% BSA, and washed again. After immunolabeling, cells were incubated with NucBlue™ Fixed Cell ReadyProbes™ Reagent (DAPI) (Invitrogen; R37606) according to the manufacturer’s protocol and imaged with the Leica DMi8 microscope (Leica Microsystems, Wetzlar, Germany) in the Leica Application Suite X 3.7.4.23463.

### 4.11. Cell Death Assay—Ferroptosis Screening

Sytoxgreen (Invitrogen, S7020) was used to quantify cell death in all 3 cell lines (MCviPI mutant, MSssI, and MCviPI) after treatment with varying concentrations of ferroptosis inducer RSL3 (0–20 µM, 1:2 dilution steps) according to the manufacturer’s protocol (n = 3 biologically independent samples per cell line). Briefly, cells were seeded at a density of 5 × 10^5^ cells/well in a 96-well plate and treated with RSL3 for 24 h. Afterwards, SYTOX green was added, and fluorescence was measured at 485 nm excitation and 520 nm emission using a microplate reader (FLUOstar Omega, BMG Labtech). Statistical analysis was carried out using a two-way ANOVA test with Tukey’s correction for multiple comparisons. A *p*-value < 0.05 was considered statistically significant.

### 4.12. Seahorse

Mitochondrial respiratory function was examined using the Seahorse XFp Cell Mito Stress Test Kit (Agilent Technologies, 103010-100) according to the manufacturer’s instructions. Briefly, 8 × 10^3^ cells/well resuspended in 80 µL complete DMEM were seeded in an 8-well XFp cell culture miniplate (Agilent Technologies, 103025-100; 3 wells with untreated cells and 3 wells with cells treated for 24 h with 1 mM FFA of the same cell line). The day after the 24 h treatment, XF DMEM pH 7.4 (assay medium), supplemented with Seahorse XF Glucose (10 mM), Seahorse XF Pyruvate (1 mM), and Seahorse XF L-Glutamine (2 mM), was used to rinse the cells (60 µL of growth medium is removed, 200 µL of assay medium is added, 200 µL medium is removed, and 160 µL of assay medium is added), and the cell culture miniplate was placed into a 37 °C non-CO_2_ incubator for 45 min to 1 h prior to the assay. Next, the Seahorse was calibrated and loaded with the XFp sensor cartridge filled with 1.5 µM oligomycin (Port A), 3 µM FCCP (Port B), and 0.5 µM Rotenone/Antimycin A (Port C). Afterwards, the cell culture XFp miniplate was loaded into the Seahorse XFp analyzer (Seahorse Biosciences, Agilent Technologies), and the real-time oxygen consumption rate was measured for 1.5 h. First baseline respiration was measured (Basal OCR) prior to mitochondrial perturbation by sequential injection of 1.5 µM oligomycin (a complex V inhibitor to decrease the electron flow through ETC); 3 µM FCCP (the uncoupling agent to promote maximum electron flow through ETC); and a mixture of 0.5 µM Rotenone/Antimycin A (complex I and complex II inhibitors, respectively, to shut down the mitochondria-related respiration). The data were analyzed using Agilent Seahorse analytics. Statistical analysis was carried out using a two-way ANOVA test with Tukey’s correction for multiple comparisons. A *p*-value < 0.05 was considered statistically significant.

### 4.13. Electron Microscopy

Cells were seeded in a T25 culture flask in a humidified atmosphere (37 °C and 5% CO_2_). Reaching approximately 70% confluency, cells were trypsinized (Thermo Fisher Scientific, 25300062), and 1 × 10^6^ were pelleted and fixed in a 0.1 M sodium cacodylate-buffered (pH 7.4) 2.5% glutaraldehyde and 0.05% CaCl_2_.2H_2_O solution at 4 °C overnight. Fixative was removed, and the sample was rinsed three times with 0.1 M sodium cacodylate, pH 7.4 (Sigma-Aldrich, 6131-99-3) containing 7.5% saccharose (Sigma-Aldrich, 57-50-1) at room temperature. Next, cells were incubated for 1 h in 1% osmium tetroxide (OsO_4_) (Sigma-Aldrich, 20816-12-0). After dehydration in an ethanol gradient, cells were embedded in an EM-bed 812 resin mixture (Electron Microscopy Sciences, Hatfield, PA, USA, EMS14120). Ultrathin sections were stained with lead citrate, and samples were examined in a Tecnai G2 Spirit Bio Twin Microscope (Thermo Fisher Scientific, FEI, Eindhoven, The Netherlands) at 120 kV. Quantification of the mitochondrial morphology was performed manually by delineating mitochondria and measuring circularity, aspect ratio ([(major axis)/(minor axis)], which reflects the ‘length-to-width ratio), and surface area in Fiji [82], as described by Lam et al. [83]. Statistical analysis was carried out using a one-way ANOVA test with Dunnett’s correction for multiple comparisons. A *p*-value < 0.05 was considered statistically significant.

## Data Availability

The data presented in this study are openly available in the NCBI GEO database (https://www.ncbi.nlm.nih.gov/geo/), reference numbers GSE240988, GSE241526, and PRJNA956894.

## Supplementary Materials

The following supporting information can be downloaded at: https://www.mdpi.com/article/10.3390/ijms242216412/s1.
