# Supplementary material for: Mitochondrial GpC and CpG DNA Hypermethylation Cause Metabolic Stress-Induced Mitophagy and Cholestophagy
Source: Int J Mol Sci. 2023 Nov 16;24(22):16412. doi: 10.3390/ijms242216412 (PMC10671279; doi:10.3390/ijms242216412)
Supplement: Supplementary file 1 [file ijms-24-16412-s001.zip › ijms-2639032-supplementary.pdf]

Supplementary material

Table S1: Overview of qPCR and PCR primers used in this study.

Gene	Forward primer (5' → 3')	Reverse primer (5' → 3')
B2M	TGCTGTCTCCATGTTTGATGTATCT	TCTCTGCTCCCCACCTCTAAGT
GSTA1	GCAGACCAGAGCCATTCTCAAC	ACATACGGGCAGAAGGAGGATC
GSTA2	CTGCCCTTTAGTCAACCTGAGG	ACAAGGTAGTCTTGTCCGTGGC
NR5a2	GGCTTATGTGCAAAATGGCAGATC	GCTCACTCCAGCAGTTCTGAAG
SLC22a7	CCTTCACCACTGCCTACCTGTT	ACAGCCACACTCCATCCAGCAA
mtND1	ATACCCCCGATTCCGCTACGAC	GTTTGAGGGGGAATGCTGGAGA
mtCOX1	CGATGCATACACCACATGAA	AGCGAAGGCTTCTCAAATCA
mtCYB	AATTCTCCGATCCGTCCCTA	GGAGGATGGGGATTATTGCT
Sall3	GTTTGGGTTTGGTTTTTGTT	ACCCTTTACCAATCTCTTAACTTTC

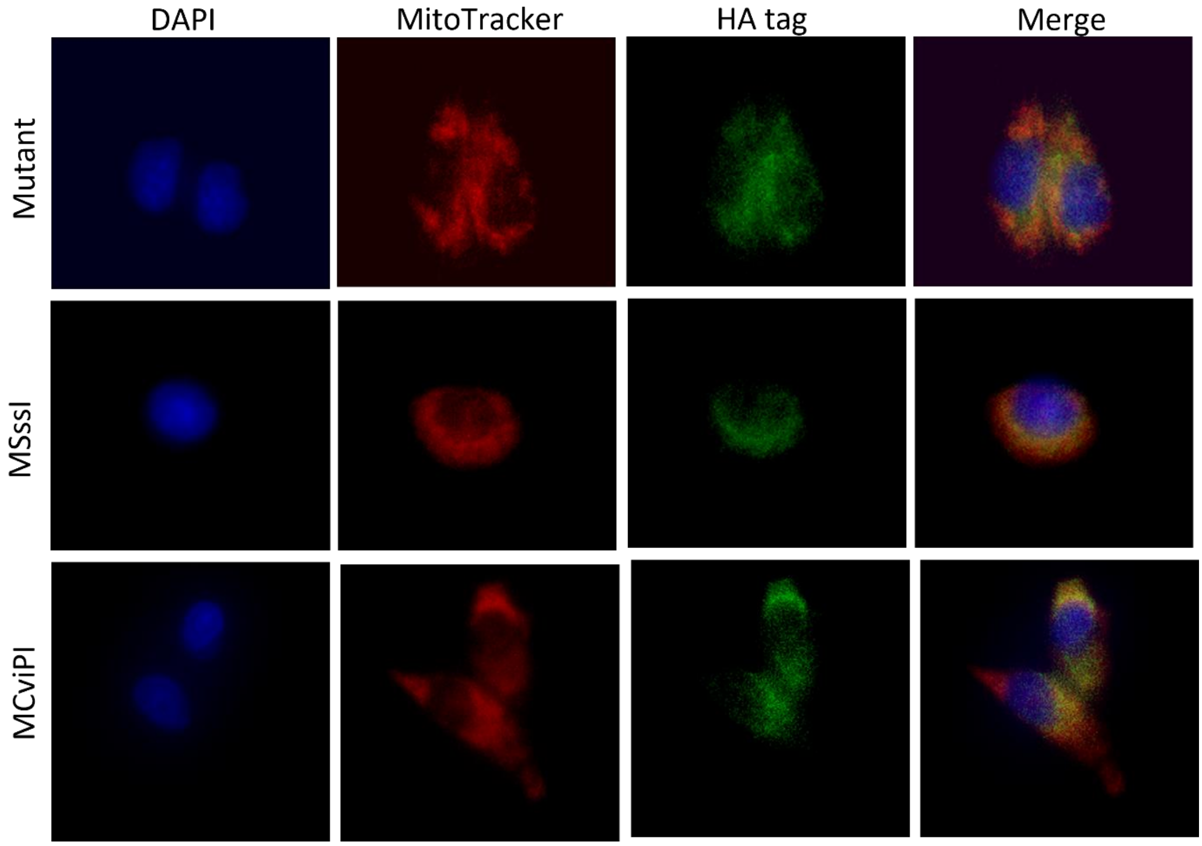

Figure S1: Colocalisation of overexpressed deficient MCviPI (Mutant), MSssI or MCviPI DNMT and mitochondria in HepG2 cells. Images showing immunofluorescence staining with anti-HA-tag targetting the overexpressed MSssI, MCviPI or MCviPI Mutant DNMT (green), the nucleus stained with dapi, the mitochondria stained with Mitotracker (red) and an overlay of these images in all three overexpressing cell lines.

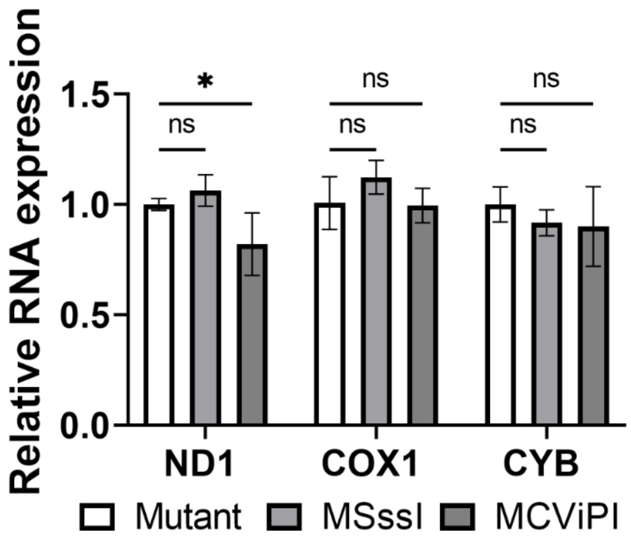

Figure S2: Mitochondrial gene expresssion. Relative mRNA expression in three different HepG2 cell lines overexpressing a GpC DNMT MCviPI (MCviPI), a CpG DNMT (MSSSI) or overexpressing a GpC/CpG DNMT deficient MCviPI mutant (Mutant). Cells were left untreated (none) (n=3 independent biological replicates). Data is shown as mean ± s.d.; (ns= not significant; * $p < 0.05$, Two-way ANOVA).

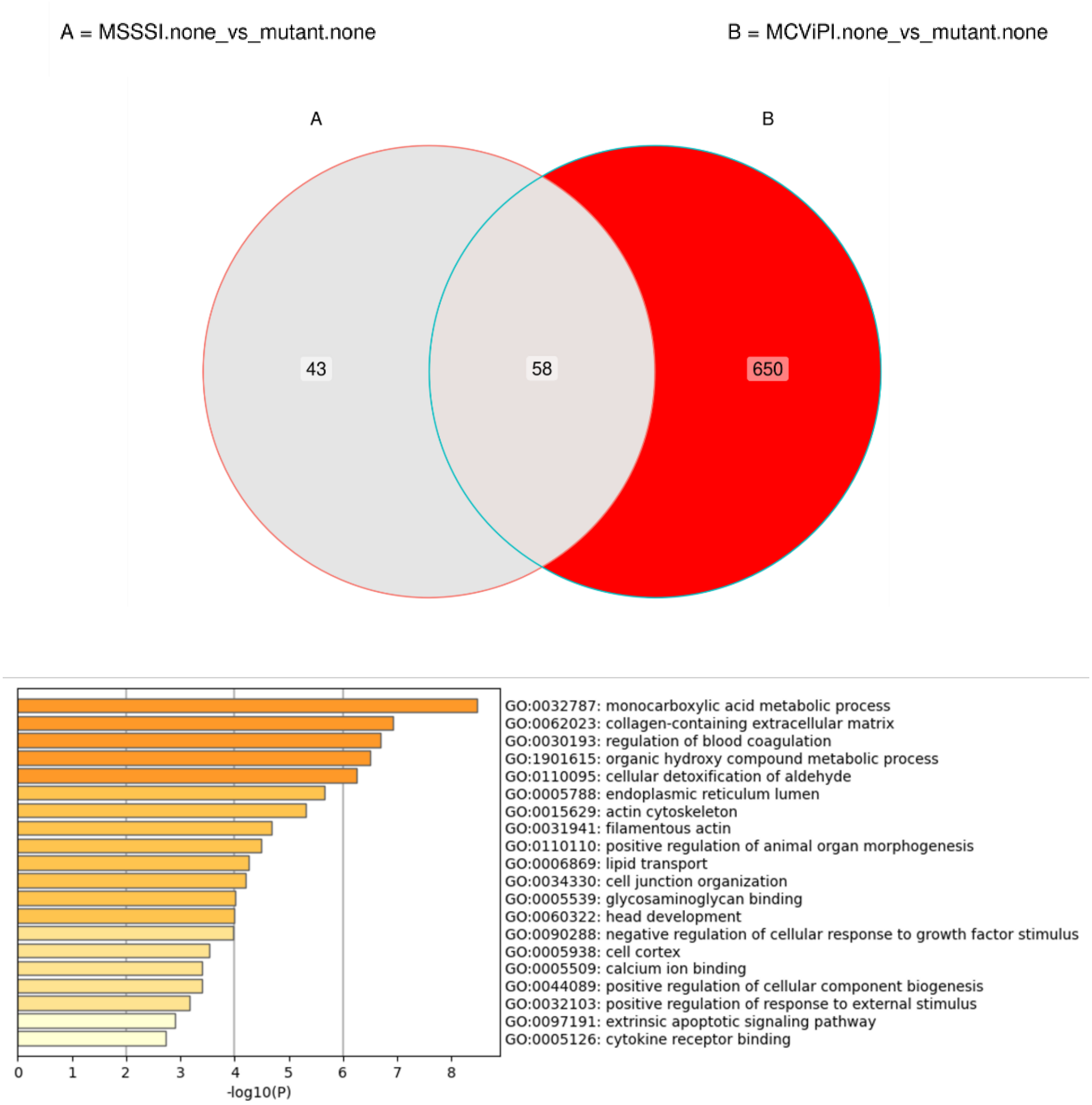

Figure S3: Differential gene expression induced by mitochondrial CpG and/or GpC hypermethylation. Venn diagram showing overlapping differentially expressed genes (FDR<0.05; log2FC >|0.2|) in the different comparisons between untreated (none) GpC MCviPI and CpG MSssI overexpressing cell lines compared to untreated control cell line MCviPI mutant (top). Metascape GO pathway analysis of the 58 overlapping genes (bottom).

Table S2: List of overlapping differentially expressed genes induced by mitochondrial CpG and GpC hypermethylation. Gene names of overlapping differentially expressed genes (FDR<0.05; logFC >|0.2|) in the different comparisons between untreated (none) GpC MCviPI and CpG MSssI overexpressing cell lines compared to untreated control cell line MCviPI mutant.

GSTA1	PLAU	ZNF678	VLDLR
GSTA2	AKR1B10	ALDH1A1	ATP10A
COL2A1	SERPINC1	IL1RAP	SERPINE2
CDH1	ADH4	NR1H4	MYL9
SPARC	SSC4D	VAV3	SLC2A1
C6	CLDN6	SAT2	CEBPD
COL16A1	SYNPO	CLK1	ASB4
UGT2B4	MYO1A	DMKN	BEX3
CES1	SULT2A1	BTG2	ABCA3
MAGED4	SULF2	CARMIL1	MATN3
CXCL8	PARD6B	FRS2	PARP16
DLX1	CCDC69	IGSF1	EDN1
HPX	NPNT	DKK1	ISM2
SMTNL2	LIPC	BAG3	
UGT2B11	PTGR1	EMILIN1	

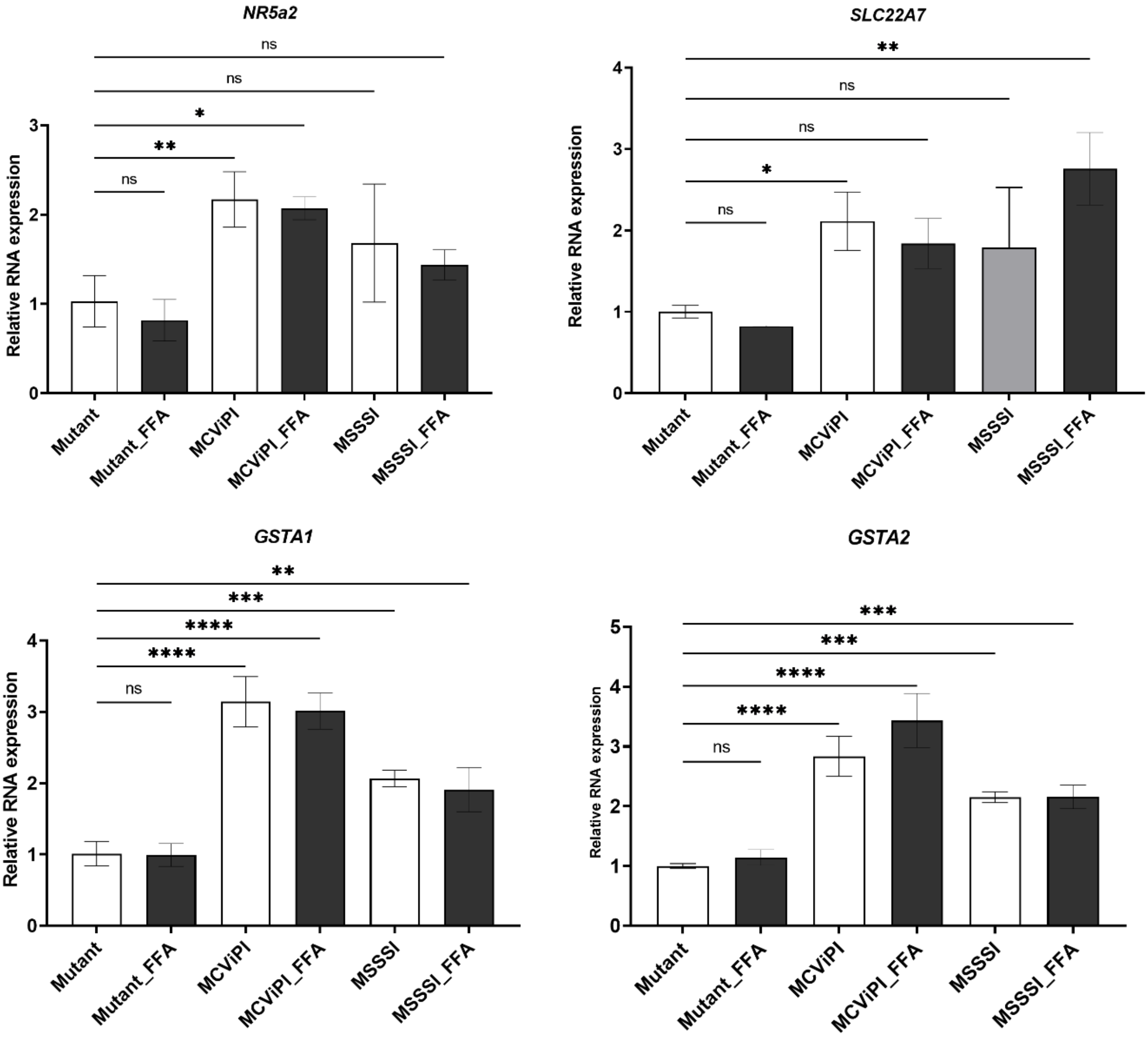

Figure S4: qPCR validation of 4 differentially expressed genes in the 3 different cell lines (MCviPI Mutant (mutant), MSssI and MCviPI) that are untreated (none) or treated with 1mM FFA for 24h. Data is shown as mean ± s.d.; n=3 independent biological replicates (ns= not significant; *$p < 0.05$, **$p < 0.01$, ***$p < 0.001$, ****$p < 0.0001$, One-way ANOVA with Tukey's correction for multiple comparisons).

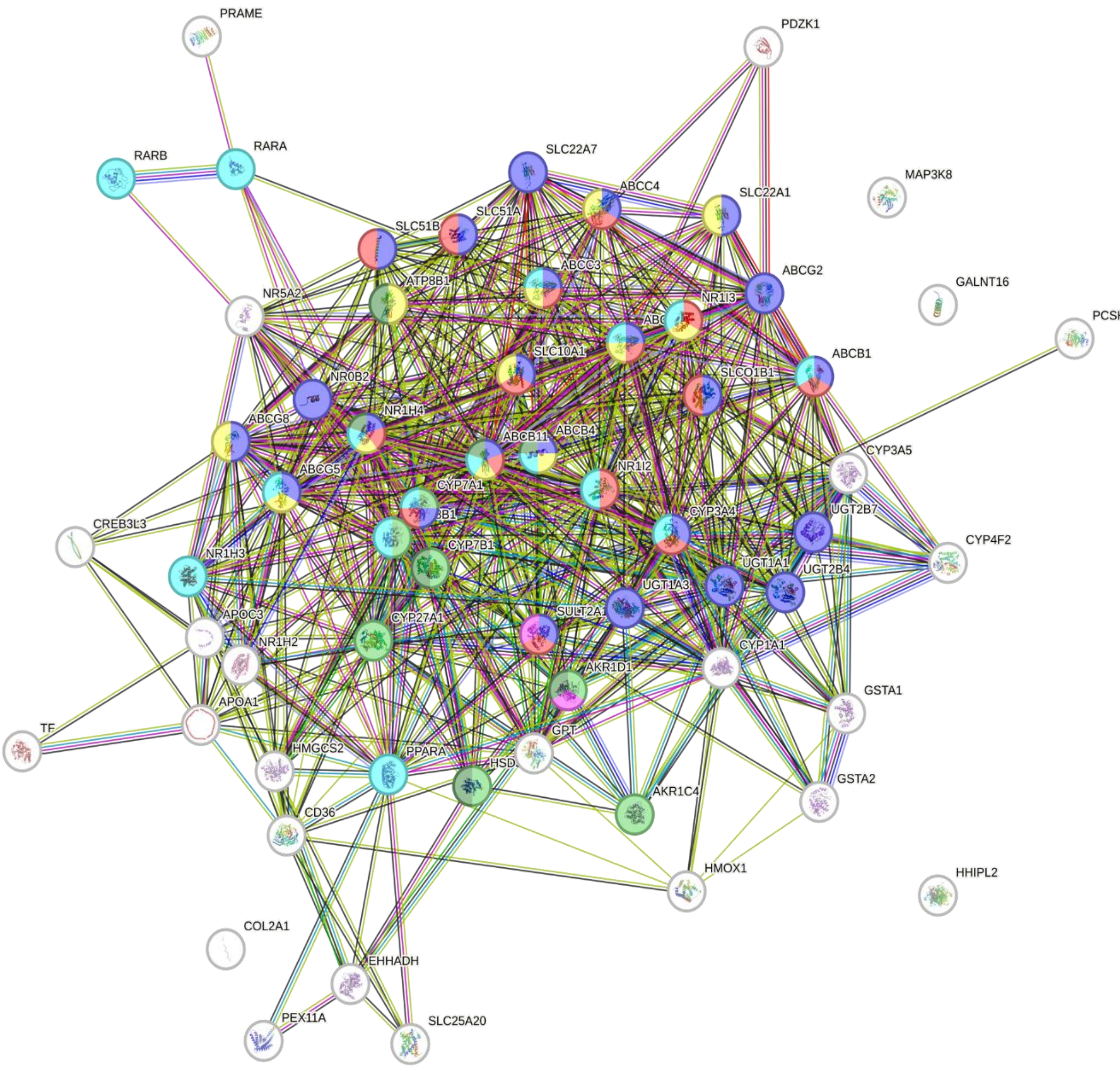

KEGG Pathways

pathway	description	count in network	strength	false discovery rate
hsa00120	Primary bile acid biosynthesis	7 of 17	2.12	3.36e-11
hsa04976	Bile secretion	24 of 88	1.94	2.38e-36
hsa02010	ABC transporters	9 of 45	1.8	6.43e-12
hsa00140	Steroid hormone biosynthesis	11 of 60	1.77	4.23e-14
hsa04979	Cholesterol metabolism	8 of 48	1.72	3.12e-10

WikiPathways

pathway	description	count in network	strength	false discovery rate
WP2289	Drug induction of bile acid pathway	15 of 16	2.47	5.06e-28
WP5238	Cholestasis	12 of 19	2.3	7.72e-21
WP5176	Disorders of bile acid synthesis and biliary transport	12 of 20	2.28	9.85e-21
WP1604	Codeine and morphine metabolism	8 of 14	2.26	1.29e-13
WP2879	Farnesoid X receptor pathway	10 of 19	2.22	8.05e-17
WP299	Nuclear receptors in lipid metabolism and toxicity	16 of 32	2.2	5.82e-27

Annotated Keywords (UniProt)

keyword	description	count in network	strength	false discovery rate
KW-0088	Bile acid catabolism	2 of 2	2.5	0.0020
KW-0988	Intrahepatic cholestasis	7 of 10	2.35	1.50e-11
KW-1278	Translocase	8 of 69	1.57	1.75e-08
KW-0752	Steroid biosynthesis	5 of 43	1.57	2.43e-05
KW-0753	Steroid metabolism	10 of 98	1.51	2.95e-10

Figure S5: Protein network of MCviPI-specific differentially expressed genes and bile acid metabolic genes. Protein-protein interaction network of differentially expressed genes in HepG2 cells overexpressing a GpC DNMT MCviPI (MCviPI) compared to HepG2 cells overexpressing a GpC/CpG DNMT deficient MCviPI mutant with genes related to cholestatis and bile acid metabolism.

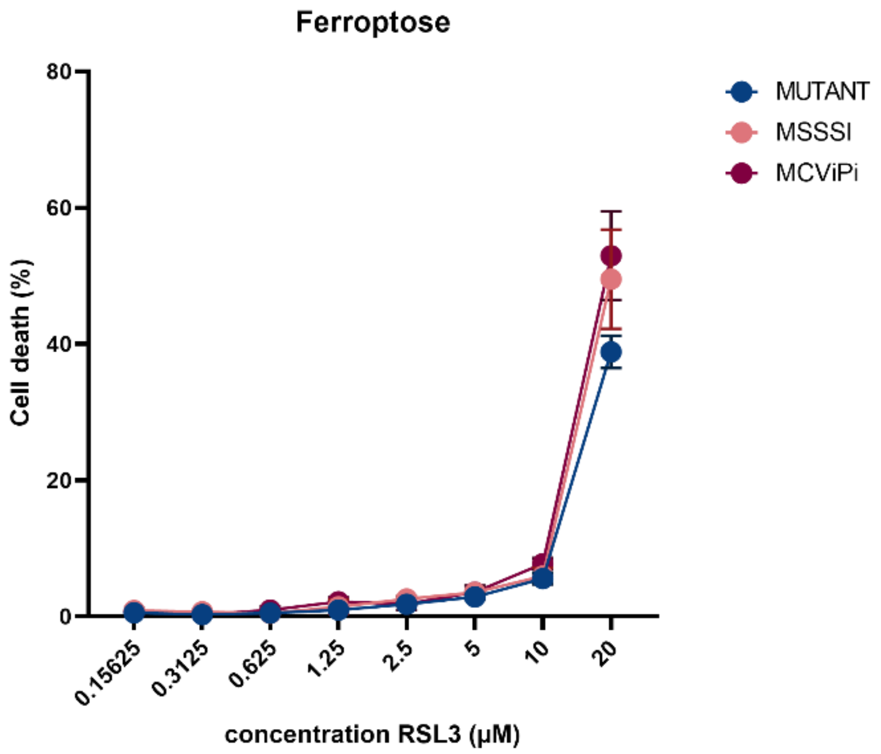

Figure S6: Ferroptosis sensitivity screening in MSssI and MCviPI overexpressing cell lines versus MCviPI mutant overxpressing cell line. Cell death assay with SYBR green after treatment with different concentrations of the ferroptosis inducer RSL3 in all different untreated cell lines. (n=3 independent biological replicates).
